# Combining Song—And Speech-Based Language Teaching: An Intervention With Recently Migrated Children

**DOI:** 10.3389/fpsyg.2018.02386

**Published:** 2018-11-28

**Authors:** Vera Busse, Jana Jungclaus, Ingo Roden, Frank A. Russo, Gunter Kreutz

**Affiliations:** ^1^English, University of Vechta, Vechta, Germany; ^2^Department of Educational Sciences, University of Oldenburg, Oldenburg, Germany; ^3^Department of Music, Speech and Music Lab, University of Oldenburg, Oldenburg, Germany; ^4^Department of Psychology, Ryerson University, Toronto, ON, Canada

**Keywords:** singing pedagogy, migrants, teaching methods, second language learning, primary school

## Abstract

There is growing evidence that singing can have a positive effect on language learning, but few studies have explored its benefit for children who have recently migrated to a new country. In the present study, recently migrated children (*N* = 35) received three 40-min sessions where all students learnt the lyrics of two songs designed to simulate language learning through alternating teaching modalities (singing and speaking). Children improved their language knowledge significantly including on tasks targeting the transfer of grammatical skills, an area largely neglected in previous studies. This improvement was sustainable over the retention interval. However, the two teaching modalities did not show differential effects on cued recall of song lyrics indicating that singing and speaking are equally effective when used in combination with one another. Taken together, the data suggest that singing may be useful as an additional teaching strategy, irrespective of initial language proficiency, warranting more research on songs as a supplement for grammar instruction.

## Introduction

Proficiency in the language of instruction is a prerequisite for learning. Enabling newly arrived migrant students to rapidly learn the language of instruction is crucial for academic achievement in the long run (Stanat and Christensen, 2006; OECD, 2010) and gains particular importance against the backdrop of current migratory movements in Europe. Refugee children often need special support, as their education may have been interrupted or post-poned due to conflict in their home countries; they may also have experienced loss and trauma, which can influence their learning behavior (e.g., Joshi and O'Donnell, 2003). Finding appropriate strategies to support second language (L2) learning while at the same time meeting the emotional and motivational needs of young migrants, who are particularly vulnerable, is therefore crucial. One way of addressing these challenges may be to use singing in addition to or in combination with traditional teaching strategies. Singing can have a positive effect on well-being (Livesey et al., 2012) and is generally advocated as an effective supplementary strategy to support language learning processes (Medina, 1993; Schoepp, 2001; Hancock, 2013). There is, however, very little empirical evidence to support these recommendations. The current study addresses this research lacuna and presents results from an intervention study where singing was used in combination with traditional teaching methods.

### Effects of music on non-musical areas of learning

It has been noted that music education is often neglected at schools (Russell-Bowie, 2009). The latter may not only be detrimental to children's musical development, but may also have negative implications for student's academic development in general. Growing evidence suggests an interrelationship between music and other academic domains including language learning (e.g., Patel, 2003; Schön et al., 2008; Moreno et al., 2009), and academic achievement in other non-musical areas of learning (Vitale, 2011). For example, primary school children who learn to play musical instruments show positive long-term effects on auditory working memory and verbal memory (Roden et al., 2012, 2013, 2014), as well as on social behavior (Roden et al., 2016). Systematic reviews looking at the effect of music-training on academic achievement tend to draw cautious conclusions (Jaschke et al., 2013; Sala and Gobet, 2017), but Sala and Gobet (2017) found a small overall effect size (*d* = 0.16) and a greater average effect size (*d* = 0.35), when only memory-related outcomes were considered.

### Effects of singing on language learning

Although there are to date few studies exploring the benefit of singing for language learning, research results have been promising. For example, when investigating Spanish-speaking children in Guatemala learning English as a foreign language (FL), Good et al. (2015) found that students taught via singing showed more improvement in English vocabulary and pronunciation than students taught via speech-based methods. Remarkably, the advantage of song over speech persisted after a 6-month delay between training and re-testing. Ludke et al. (2014) applied a listen-and-sing-strategy in native English-speakers to facilitate FL learning (Hungarian). Learning was also significantly more successful in the singing condition even when controlling for several cognitive variables. These studies also resonate with an earlier study by Salcedo (2010), wherein learners recalled significantly more text after singing than after reading a text passage.

The song-advantage for language learning could be based on different mechanisms. Firstly, the use of temporal (rhythm, tempo) and pitch variation (melody) in song may offer enhanced psychoacoustic cues that facilitate the encoding and/or retrieval of to-be-learnt materials in laboratory studies (Calvert and Tart, 1993; Wallace, 1994; Rainey and Larsen, 2002; Ludke et al., 2014). In general, the rate of text presentation in singing has been found to be ~3-quarters of the rate found in speech (Kilgour et al., 2000). In studies involving students learning text in their native language where the rate of text presentation was equated, the song advantage disappeared (Kilgour et al., 2000; Racette and Peretz, 2007). Therefore, one factor that may contribute to the song-advantage might be the relative duration (with the implication of differing presentation rates) of sung vs. spoken text. However, genuine song-advantages could be found in studies involving FL learners when controlling for text presentation rate (Ludke et al., 2014; Good et al., 2015). Thus, it appears that a song-advantage exists in FL learning even when extraneous variables are controlled for.

Secondly, musical activities are generally perceived as enjoyable, thus enhancing positive affect and motivation (Kreutz et al., 2004). The latter are particularly important in FL/L2 learning. Opposed to first language (L1) acquisition, the FL/L2 learning process may have many ruptures and regressions (e.g., Busse, 2014), and it is therefore not surprising that positive affect and motivation have proven to be crucial factors influencing language achievement (Dörnyei, 2005, see also the meta-analysis by Masgoret and Gardner, 2003).

Finally, group singing can positively influence cooperation and empathy in children from pre- (Kirschner and Tomasello, 2010) to primary school age (Good and Russo, 2016). In the latter study, children from a summer camp were randomly assigned to three groups, namely a competitive sport games group, an arts group, and a singing group. After the intervention, all children played several rounds of a child-adapted version of the prisoner's dilemma game as a proxy for empathy. Children who were assigned to the singing group showed significantly greater levels of empathy in comparison to the other groups (Good and Russo, 2016).

### Limitations of existing research and implications for current study

Although there is already growing evidence of the relevance of singing for instruction at school, there are also important limitations to previous studies. For instance, studies focus on L1 or FL learners thus neglecting L2 learners, who by nature form very heterogeneous groups because of different language backgrounds and previous language knowledge^1^. At the same time, promoting well-being and creating an inclusive, cooperative learning group in addition to teaching learning content is particularly important when teaching L2 learners, including young refugees. In addition, studies exploring the effect of song on language learning mostly focus on memorizing text passages or new words, phonological awareness, and pronunciation, while grammar skills seem less frequently addressed (Alinte, 2013). Despite the wealth of literature recommending music and songs to develop literacy and grammar skills (e.g., Brown, 2006; Paquette and Rieg, 2008; Hancock, 2013), empirical evidence is scarce. One may hypothesize that sound may also be less important for teaching grammar than for teaching pronunciation, given its more conceptual character. More studies are needed in order to assess to what extent singing is beneficial for stimulating grammar skills, and whether these skills are also demonstrated on transfer tasks.

In general, using singing for grammar teaching may be particularly suitable for primary school children. Firstly, younger learners usually show positive responses to group singing (Good and Russo, 2016) and may not be inhibited by voice breaks or other maturation effects. Secondly, although there is evidence to suggest that even younger learners can benefit from a combination of implicit and explicit teaching (Stanat et al., 2012), in general explicit grammar teaching (e.g., explanation of grammar rules) may be more difficult for younger learners (Cameron, 2001, S. 106; Cummins, 1981). Interestingly, when students learn an FL in the school setting, older learners tend to outperform younger learners (e.g., Muñoz, 2010; Jaekel et al., 2017). This advantage may be attributed to greater levels of cognitive maturity, which may help them to better process grammar explanations (also see Cummins 1981). In addition, older FL learners usually have better developed literacy skills in their L1, which they can draw on when learning another language (Pfenninger, 2016). It may therefore be assumed that implicit grammar teaching (e.g., through songs designed to highlight certain grammatical phenomena) is well suited for younger learners (Dekeyser, 2008; De Graaff and Housen, 2009).

The current study looks at the role of singing as a complementary language teaching strategy in the L2 classroom. The classrooms studied were composed of recently migrated students in German primary schools. Both modalities were employed in each session, with one song taught through singing and the other through speaking. The outcomes of interest were the expansion of vocabulary and the development of grammatical skills.

## Research questions and hypotheses

The overall aim of the current study is to explore the effect of an intervention on L2 learning where singing was used as a complementary teaching strategy. The study addresses the following research questions:

1. *To what extent does a short intervention comprising three teaching intervals (40 min. each) promote L2 learning?* We expected significant gains on the language knowledge test between baseline (t1) and post-test (t2) taken after the intervention, and that these gains would be sustained in the follow-up test (t3) over a retention period of 3–4 weeks. We also expected gains in tasks requiring students to transfer grammatical skills to unknown words (H1).
2. *To what extent does intervention-based learning of songs increase vocabulary recall and grammatical accuracy?* One could expect differential effects of speech- vs. song-based teaching with an advantage of song-based teaching. In particular, we expect students to recall songs more accurately when taught through a song-based approach compared to a speech-based approach (H2).
3. *What is the impact of students' prior language knowledge on learning outcomes?* In general, we expect students with more prior language knowledge to demonstrate better learning outcomes than learners with less prior language knowledge. However, we expect learners with less prior knowledge to make similar learning gains to those with more prior language knowledge (H3). We also explore to what extent students' prior knowledge might moderate the effectiveness of teaching modalities and identify any children who may not respond to the teaching intervention or respond particularly well to it.

## Methods

### Participants

The sample consisted of primary school learners who attended two publicly funded state schools in urban areas of Lower Saxony and who were recruited from different classes for the intervention. All learners, including refugees, had recently migrated to Germany. In fact, many of the students had not yet been settled in the community and were still living in refugee camps. Languages spoken by children were Arabic, Kurdish, Turkish, and Farsi. In total, the sample comprised 35 learners (18 girls, 17 boys) with an age range from 6 to 11 (*M* = 8.46, *SD* = 1.58). Teachers judged these recently migrated students to be very heterogeneous; they observed strong differences in learning progress and felt that many struggled with short attention spans; some of them also displayed discipline problems.

The study was conducted in accordance with the recommendations of the *Carl von Ossietzky University's Ethics Committee*. This committee approved the protocol of the current study. All parents of participant students received information about the project (translated into their respective languages) and gave written informed consent in accordance with the Declaration of Helsinki. In addition, all children were informed orally about the study, and the procedures were explained.

### Design and intervention

A quasi-experimental intervention study with a pre-post- and follow-up design was conducted to answer the research questions. Recently migrated children were taught the text of two songs through two teaching modalities (singing/speaking) in three small groups ranging from 10 to 15 students. Two groups (23 children) sang Song A and spoke Song B (henceforth collectively described as Group A), and one group (12 children) sang Song B and spoke Song A (henceforth described as Group B); in each session, singing and speaking was combined. One school decided at short notice that students could not participate, which led to a different number of participants per modality (see Table 1).

**Table 1:** Sample size by modality (*N* = 35).

**Song**	**Modality**	
	**Sung**	**Spoken**
Song A	23	12
Song B	12	23

**Figure 1:**
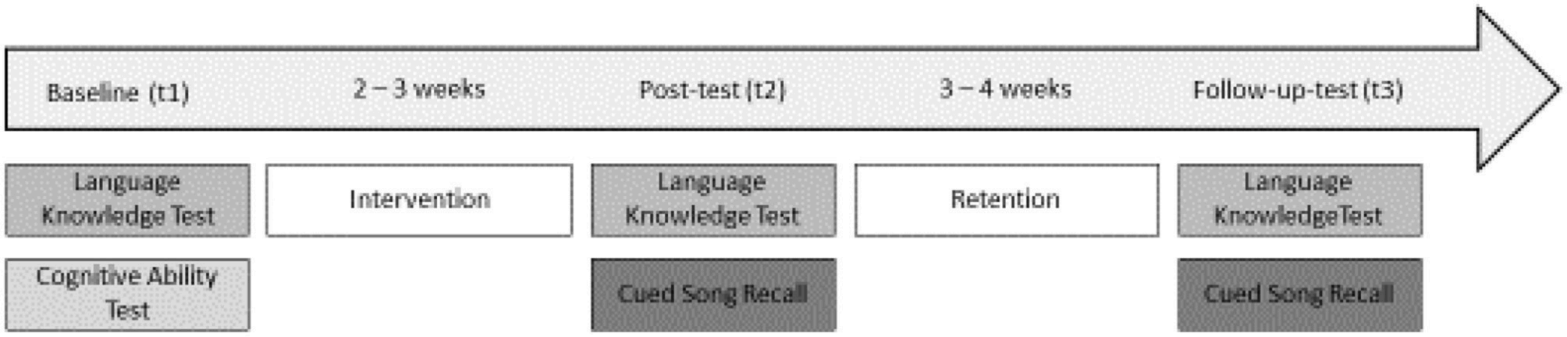
Overview of study design.

At time 1 (t1), which was the baseline, a cognitive skills test and an intervention based language knowledge test which assessed song-based vocabulary and grammatical skills were conducted (see “*Materials*”). After the intervention (t2), the language knowledge test was administered again. In addition, students' ability to recall the songs was tested (cued song recall). Three to four weeks later (t3), the language knowledge test was administered a third time and cued song recall was tested again (follow-up). Given the short duration of the intervention, the retention interval was designed to be longer than the intervention itself in order to ensure that developmental effects or effects of test repetition would only marginally influence changes in test scores (see Figure 1).

### Materials

Three four-line stanzas from two songs *Wer mag wen*? (Song A *Who likes whom*?) and *Mitmachlied* (Song B *Join in song*) were selected for the intervention. Selected songs were specifically composed to support early literacy and grammar skills of primary school children (Fuchs and Röber, 2008). The songs were entirely novel to participants prior to the study.

The lyrics of Song A contained 57 words in total of which 21 words (without the corresponding articles for nouns) were selected for testing. Song B contained 72 words of which 36 were selected for testing. We chose verbs and nouns, because they target specific grammatical areas that students had low command of. Song A's practice words included male and female job titles such as the word farmer, which is modified from *Bauer* (masculine) to *Bäuerin* (feminine). The verb *mögen (to like)* is conjugated into its first, second and third person singular forms (*ich mag, du magst, er mag*). Song B targeted singular and plural noun forms (*Baum-Bäume; tree - trees*). The plural form of all nouns in the material contain umlauts and the same ending, allowing students to practice forming the plural (*Kopf-Köpfe*; head—heads). Students also practice verb conjugations (*ich wackle—wir wackeln; I wag-we wag)*. This is particularly difficult in combination with case inflection. Additionally, both dative (*wackeln mit dem Kopf*; wag with the head) and accusative (*klopfen an die Wand; knock on the wall*) prepositions are used in the song. The song material can be found in Appendixes A,B with target words chosen for testing (nouns and verbs only) in bold. As the two songs differ in musical criteria, and linguistic content (grammatical areas targeted), we analyse recall of lyrics for each song separately.

### Dependent variables

Based on the songs, a language test was developed to assess vocabulary and grammatical skills of participants. Some items tested knowledge of the words (nouns and verbs) that were introduced through the songs, other items looked at knowledge of verb conjugations, the ability to produce both plural and feminine forms of nouns. Some items tested whether students could apply the grammatical rules to unknown substantives (transfer items). Retest reliabilities of language knowledge test tasks ranged from *rtt* = 0.49 to 0.81. Intercorrelations within and between the three time points can be found in Appendix C. In addition, exact recall of the songs was tested. For Song A, the theoretical maximum consisted of 21 words; for Song B, the theoretical maximum consisted of 36 words. Retest reliability for cued recall tests ranged from *rtt* = 0.75 (Song A) to *rtt* = 0.54 (Song B).

### Independent variables

The German adaptation of Cattell's Culture-Fair Intelligence Test (Cattell, 1961) by Weiß and Osterland (2013), CFT-1R was administered at baseline to measure fluid intelligence using its six subtests: substitutions, mazes, similarities, series, classifications, and matrices. According to (Weiß and Osterland, 2013) the CFT-1R correlates highly with the “g”-factor of intelligence (*r* = 0.66). Results were assessed by raw means for each of the six subtests. As reported in the manual, retest reliabilities of all six subtests ranged from *rtt* = 0.63 to *rtt* = 0.86. The test is based on non-verbal visual puzzles and designed in a way that it can largely be administered without verbal input.

### Procedure

To ensure high ecological validity, the intervention was conducted at the respective schools attended by the recently migrated school children. Intervention length was constrained by the time obligations of participating schools.

First, the cognitive skills test and the intervention based language knowledge test were administered to all participants in a school classroom (t1). Instructions were given in German. Trained test administrators carried out the assessments according to the instructions of a detailed manual and script. Data collection at t1 took place on 2 different days. First, students completed the cognitive skills test, then students completed the language knowledge test.

In the following 2 weeks, the learning unit was implemented during three 45 min. sessions during regular school hours. The intervention consisted of two phases, one singing phase (20 min.), and one speaking phase (20 min.), the order of which were alternated after each lesson. In each lesson, students also completed a very brief questionnaire looking at physical and emotional well-being (*I feel well/I feel tired*) before and after the intervention (5 min.). About half a week after the intervention (t2), the language knowledge test was re-administered to all participants. In addition, an assessment was made regarding the extent to which students were able to remember the lyrics of the songs they had been taught (cued song recall). For cued song recall, children were tested individually in a separate room. All children were asked to recall as much as they could of the song lyrics verbatim without the support of testers. Once a child had exhausted their recall, a tester provided a short prime by reproducing the title of each song and short trigger words (e.g., der *blank* mag die *blank*). Children were not given specific instruction about whether they should sing or speak the lyrics. The tester transcribed the words in the same way children pronounced them regardless of correctness. Full points were awarded when the child recalled the word in its correct form and pronounced the umlaut correctly. Half a point was awarded when the child recalled the correct form of the word but did not pronounce the umlaut or feminine ending correctly. E.g., if students did not pronounce the umlaut in words such as *B***ä***uerin*, the testers wrote down *B****a****uerin* and the answer was awarded half a point. No points were awarded if words were not remembered at all or if the pronunciation deviated in aspects other than umlaut or endings. In order to assess significant differences between correct recall of grammar and vocabulary recall in general, there was a second coding which ignored grammatical correctness (e.g., full points even if endings/or umlauts were incorrect). All results were double-checked by a second assessor who detected no errors. During the retention interval, students attended regular school lessons. No further language tuition interventions took place. Care was taken that the intervention songs were not used by teachers in their lessons.

Three to 4 weeks later (t3), students were administered the language knowledge test a third time and the cued recall test was conducted again (follow-up). The learning unit was implemented according to a detailed script by two pre-service teachers with a BA degree in music education. Teachers also received intercultural training as part of the protocol for a previous project (Busse and Krause, 2016) and could therefore be expected to be sensitive to cultural issues.

The learning and cued-recall procedures were modeled on Good et al.'s 2015 intervention study. One deviation that was necessary concerned the provision for translation of song lyrics to native text. This aspect of the design was not feasible due to variability in native languages. Instead, during the learning sessions, the teachers presented each line of the lyrics along with pictures and symbols above each line to help students understand the meaning of the songs. Only one of the song's three stanzas was introduced in each lesson. The first exposure of the stanza was always presented without interruption, allowing the children to hear it in its entirety. Subsequent exposures involved breaking each phrase down into four (Song A) or six (Song B) sections. A point of melodic and metric closure between the sections in the sung phrases allowed for a natural separation of the sections. The same division was used when the lyrics were spoken. Teachers employed the “repeat-after-me” method, stopping at the end of each section in order for students to repeat back the lyrics. Lines were then repeated in their entirety.

To minimize variability beyond the main experimental manipulation, each learning session provided exactly 20 min of exposure per modality. In addition, speed of presentation and the number of repetitions were controlled. Teachers were instructed to slow down their natural rate of speech in order to adjust for the natural discrepancy in tempo between spoken and sung speech.

### Data analysis

Explorative data analysis suggested that language knowledge results were normally distributed, allowing for the use of parametric statistics. In order to explore the overall effect of the intervention on language test results (RQ1), we conducted a one-way ANOVA with repeated measurement, comparing t1, t2, and t3. Preconditions for conducting ANOVAs were tested (normality, Box's M test of equality of covariance matrices, Mauchly's test of sphericity) and were met in all instances except for cued song recall, where data was not normally distributed. In order to explore the effect of intervention-based learning of songs on vocabulary recall and grammatical accuracy (RQ2), we therefore additionally conducted non-parametric statistics. There was no difference between parametric and non-parametric results except in one case, where we report results from non-parametric statistics (Wilcoxon Signed-Ranks test), as the data were not normally distributed and results from non-parametric statistics are therefore bound to be more reliable. To explore the impact of students' prior language knowledge on learning outcomes (RQ3), a median split was performed, and students were divided into two groups based on higher and lower prior language knowledge at baseline results (t1). A two-way repeated measures ANOVA was used to explore the effect of time and language knowledge test results with groups divided according to prior language knowledge (high vs. low). In addition, a two-way ANOVA was used to compare the impact of prior language knowledge and teaching modality on cued song recall at t2. All analyses were conducted with SPSS version 24.

## Results

### RQ1: To what extent does a short intervention comprising three teaching intervals (40 min. each) promote sustainable L2 learning?

Language proficiency as assessed in the language knowledge test, geared at both vocabulary and grammar skills, increased significantly over time. Specifically, at t1 (baseline), students reached 60.38% of the theoretical maximum score, whereas after the intervention at t2, they reached 71.13%. Learning was also sustainable over the retention interval, and students reached 71.97% at t3. Table 2 shows descriptive data for total scores and additionally for the two groups. A one-way repeated measures ANOVA with the factor “time” revealed a significant large effect [Wilks' Lamdba = 0.38, *F*_(2, 31)_ = 25.74, *p* < 0.0001, $n_{p}^{2}$ = 0.62].

**Table 2:** Performance language knowledge test (max. 32): means and standard deviations for both groups at the three time points.

	Group A^*^	**Group B**	**Total**
	***M* (*SD*)**	***M* (*SD*)**	***M* (*SD*)**
Language knowledge t1	19.50 (5.62)	19.00 (6.66)	19.32 (5.92)
Language knowledge t2	22.81 (5.63)	22.42 (7.04)	22.76 (6.01)
Language knowledge t3	22.55 (5.94)	23.63 (6.19)	23.03 (5.87)

* *Group A sang song A and spoke song B, Group B sang song B, and spoke song A*.

**Figure 2:**
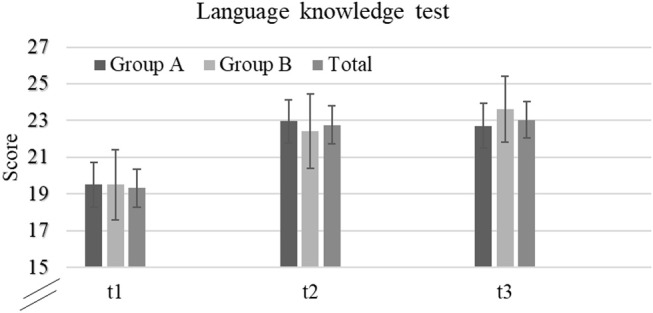
Performance language knowledge test (max. 32): means and standard deviations for both groups at the three time points.

We also assessed transfer skills by looking at progress on those items that required transfer of grammatical skills to unknown words. Students scored higher at t2 (*M* = 6.11, *SD* = 2.45) and at t3 (*M* = 6.02, *SD* = 2.78) than at t1 (*M* = 5.00, *SD* = 2.42). A one-way repeated measures ANOVA with the factor “time” also revealed a significant large effect [Wilks' Lamdba = 0.58, *F*_(2, 31)_ = 11.21, *p* < 0.0001, $n_{p}^{2}$ = 0.42]. It thus appears that despite its brevity, the intervention promotes sustainable L2 learning, not only in terms of expansion of vocabulary but also in terms of grammar development. H1 was therefore confirmed (see also Figure 2).

### RQ 2: To what extent does intervention-based song-learning increase vocabulary recall and grammatical accuracy?

Students in the two groups were comparable as regards their cognitive abilities and language knowledge at t1. An independent samples *t*-test comparing the two groups at t1 revealed no differences regarding either cognitive abilities [*t*_(31)_ = 0.60, ns] or regarding language knowledge test results [*t*_(31)_ = −0.23, ns].

Language gains as assessed through cued recall of both songs show that students recalled over 17% of Song A (t2: 17.44%; t3: 25.84%; see Table 3) and over 15% (t2: 15.61%; t3: 19.65%; see Table 4) of Song B. A Wilcoxon Signed-Ranks test indicates that there was no significant difference between t2 and t3 neither for Song A [*Z* = −2.67, ns] nor for Song B [*Z* = −0.77, ns]. Note, that high standard deviations suggest strong variability within groups, which will be discussed below (see RQ3).

**Table 3:** Performance cued recall of Song A (max. 21): means and standard deviations for both conditions and time-points.

	**Sung**	**Spoken**	**Total**
	***M (SD)***	***M (SD)***	***M (SD)***
Cued recall t2	3.50 (5.41)	3.96 (4.63)	3.66 (5.08)
Cued recall t3	5.32 (5.76)	5.63 (5.19)	5.43 (5.49)

**Table 4:** Performance cued recall of Song B (max. 36): means and standard deviations for both conditions and time-points.

	**Sung**	**Spoken**	**Total**
	***M (SD)***	***M (SD)***	***M (SD)***
Cued recall t2	7.04 (12.53)	4.84 (7.71)	5.62 (9.56)
Cued recall t3	10.42 (12.98)	5.25 (8.68)	7.07 (10.51)

In order to asses to what extent intervention-based song-learning increases language skills, we first compared cued song recall results with respect to the two teaching modalities. Repeated measures ANOVA with the factors “time (t2 and t3)” and “cued song recall results” regarding Song A revealed a significant effect for time [*F*_(1, 32)_ = 6.64, *p* = 0.02, $n_{p}^{2}$ = 0.17] and no significant group effect [*F*_(1, 32)_ = 0.05, ns]. There was also no significant interaction effect [*F*_(1, 32)_ = 0.01, ns]. Regarding Song B, there was no significant effect for time [*F*_(1, 32)_ = 1.19, ns] or group [*F*_(1, 32)_ = 1.37, ns] and no significant interaction effect either [*F*_(1, 32)_ = 0.73, ns]. In other words, students did not recall songs more accurately when taught through a song-based approach thus contradicting H2.

In order to find out whether there were differences between vocabulary recall and grammatical accuracy, we also coded cued song recall test results at t2 according to vocabulary recall (recall of words independent of grammatical accuracy) and according to grammatical accuracy (recall of correct forming of singular and plural forms, verb conjugations, case inflection, and feminine nouns). Tables 5, 6 show that differences between vocabulary recall and grammatical accuracy were only small.

**Table 5:** Performance cued recall of Song A (max. 21): means and standard deviations for vocabulary learning and grammatical accuracy at t2.

	**Sung**	**Spoken**	**Total**
	***M (SD)***	***M (SD)***	***M (SD)***
Recall of vocabulary	3.64 (5.61)	4.25 (4.79)	3.85 (5.27)
Grammatical accuracy	3.41 (5.31)	3.67 (4.54)	3.50 (4.98)

**Table 6:** Performance cued recall of Song B (max. 36): means and standard deviations for vocabulary learning and grammatical accuracy at t2.

	**Sung**	**Spoken**	**Total**
	***M (SD)***	***M (SD)***	***M (SD)***
Recall of vocabulary	6.17 (11.46)	5.00 (7.90)	3.85 (5.27)
Grammatical accuracy	5.75 (11.04)	4.59 (7.31)	3.50 (4.98)

A two-way repeated measures ANOVA with the factors “modality” and “language gains (vocabulary vs. grammar) in cued song recall” revealed a significant effect of the factor “language gains” [Song A: *F*_(1, 32)_ = 6.10, *p* = 0.02, $n_{p}^{2}$ = 0.16; Song B: *F*_(1, 32)_ = 4.68, *p* = 0.04, $n_{p}^{2}$ = 0.13]. There was, however, no significant effect of the factor “modality,” and results showed no difference regarding teaching modality [Song A: *F*_(1, 32)_ = 1.18, ns; Song B: *F*_(1, 32)_ = 0.13, ns]. There was no interaction effect either [Song A: *F*_(1, 32)_ = 1.18, ns, Song B: *F*_(1, 32)_ < 0.0001, ns]. In other words, the difference between vocabulary recall (recall of words independent of grammatical accuracy) and grammatical accuracy (recall of correct forming of singular and plural forms, verb conjugations, case inflection, and feminine nouns) was significant but not very pronounced and children often remembered words in the correct grammatical form. The teaching modalities (singing and speaking) proved equally effective thus contradicting H2.

### RQ 3 what is the impact of prior language knowledge on learning outcomes?

Students with high prior language knowledge test results scored higher on the language knowledge test at t2 and t3 (see Table 7). Repeated measures ANOVA with the factors “time” and “language knowledge test results” with groups divided according to prior language knowledge (high vs. low) revealed a significant effect for time [*F*_(2, 30)_ = 25.56, *p* < 0.0001, $n_{p}^{2}$ = 0.63] with a large effect size, and for language knowledge test results [*F*_(1, 31)_ = 79.07, *p* < 0.0001, $n_{p}^{2}$ = 0.72]. However, the interaction effect was not significant [*F*_(2, 30)_ = 1.08, ns]. Hypothesis 3 was therefore confirmed, as students with more prior language knowledge also demonstrate better learning outcomes than learners with less prior language knowledge at t2 and there was no language knowledge-treatment interaction; i.e., students with high and low prior language knowledge seemed to equally benefit from the intervention.

**Table 7:** Performance baseline- and post-language test tasks (max. 32): means and standard deviations.

	**Group low prior language knowledge (*n* = 17)**	**Group high prior language knowledge (*n* = 16)**
	***M (SD)***	***M (SD)***
Language knowledge t1	14.44 (3.27)	24.50 (2.77)
Language knowledge t2	17.91 (4.08)	27.72 (2.85)
Language knowledge t3	18.82 (4.74)	27.31 (3.41)

*Low (≤19.5) and high (>19.5) indicate two levels of prior language knowledge based on median split*.

In order to explore whether there were any children who may not respond to the teaching intervention, we also identified students who did not make any progress on the language knowledge test or who failed to recall the song lyrics. Regarding the language knowledge test, there were only five students who did not make any progress; these students also had difficulties recalling the songs. Two of these students had high initial language knowledge test results, which remained unchanged; three students had little prior knowledge. Regarding cued song recall, we identified 15 students who did not recall the song lyrics. Twelve of them had low prior language knowledge test results, but three students did not make any progress despite above-average prior language knowledge. We also identified seven students in the cued song recall test who remembered large parts of the songs, with one student recalling the lyrics entirely, both at t2 and t3. Except for one student, these students had above average prior language knowledge. The lyrics recalled extensively were all taught via singing.

## Discussion

Learning the language of instruction is one of the major challenges for recently migrated students. The overall research programme seeks to support recently migrated students by promoting language learning as well as psychological well-being at school through a music-supported intervention. The study presented here was explorative in nature and examined the effectiveness of an intervention which used song as an additional teaching strategy to facilitate language learning. Unlike previous studies (e.g., Good et al., 2015), the intervention aimed to develop grammar skills in addition to vocabulary. The results indicate that the intervention itself was successful. On average, students made significant learning progress on the language knowledge test, which assessed song-based vocabulary, and grammatical skills. Importantly, children also showed progress on tasks that required transfer of grammatical skills. The large effects observed suggest that children were able to abstract rules and apply them to unknown words. Importantly, learning effects were also sustainable over a period of 3–4 weeks. Results are encouraging given the brevity of the intervention and the challenges presented in the sample (i.e., heterogeneous groups, short attention spans and behavioral problems of some students). Language gains as assessed through cued recall of both songs shows that students recalled on average of 15% (Song B) to 17% (Song A) of the lyrics. This represents a considerable learning gain, as students were not familiar with the songs. In addition, the lyrics introduced in the intervention were cognitively more demanding (introducing not only new words but also new grammar structures) and considerably longer (Song A 57 words, and Song B 72 words) than lyrics used in other intervention studies (e.g., Good et al., 2015 used one song with 31 words). When comparing text recall irrespective of grammatical accuracy vs. text in its correct grammatical form, significant differences could be observed, but these were not very pronounced in comparison with general language gains. In other words, children often remembered words in the correct grammatical forms (e.g., they did not only use an -e to form the plural of one-syllable words, but also made the umlaut change).

However, in contrast to other song-based interventions (e.g., Ludke et al., 2014; Good et al., 2015), learning progress did not seem to be differentially affected by the teaching modalities (singing vs. speaking) thus contradicting our research hypothesis. It could alternatively be assumed that the motivating effect of singing carries over to the speaking modality. The additional questionnaire data indicates that students' emotional and physical well-being remained constant during the intervention which would support this hypothesis. However, we also noted that relatively few children chose to sing or hum the melody of the lyrics they had learnt through the singing modality during recall testing which contrasts with findings in other studies (e.g., Calvert and Tart, 1993; Good et al., 2015). The fact that students were not familiar with the melody of songs prior to the study may have played a role. A recent study by Tamminen et al. (2017) found that adult participants only remembered novel words better in the sung than in the spoken modality if participants were sufficiently familiarized with the song (Tamminen et al., 2017). We similarly noted in our sample that children who chose to sing or hum the melody appeared to be more successful in retrieving the lyrics. These learners may have used the cues provided by melodic and rhythmic structure as a retrieval strategy to support recall. Future studies may therefore explore students' retrieval strategies in a more systematic manner. From a pedagogical point-of-view it may therefore be advisable to use easy and catchy melodies from songs that students are familiar with and/ or to teach the melodies before introducing them in the context of language learning.

In addition, studies of long-term memory for songs have revealed that cultural aspects (e.g., the origin and popularity of a given tune) and their functional meaning in every-day practices (e.g., celebrations, festivities, rituals) can influence their mental representations along with their structural complexity (Büdenbender and Kreutz, 2015, 2017). Songs that were developed for specific purposes such as foreign or second language learning must also be acknowledged for potentially different levels of meaning beyond their surface structures (melody and rhythm) and associated linguistic content (lyrics).

Much research has shown that there are differential effects of teaching approaches for learners with different prerequisites (see, for instance, the expertise reversal effect in Kalyuga et al., 2003). The data in the current study indicate that song can be a useful supplement even for learners with very little prior knowledge, as there was no interaction effect between prior language knowledge and treatment. However, we also identified some students who did not make any progress and who failed at song recall. It appears that most of these students also had below average language knowledge test results at baseline, but there was also a small number of students who made no progress despite above-average language knowledge test results at baseline. This may have to do with the lack of familiarity with the melody (see above) or with relatively high demands of free recall tasks in comparison to recognition. One may further hypothesize that there are other factors influencing learning progress, which we did not explore. Future studies may look at motivation prerequisites and attention during intervention in more detail.

We identified some students who benefitted particularly well from song-based teaching and recalled large parts of the lyrics taught through the singing modality. Most of these students had above average language skills at baseline. Due to the small sample sizes, we could not fully explore the possibility of differential treatment effects for such subgroups. Explorative tests suggest that these students also made significantly better learning progress on the language knowledge test; future studies with larger sample sizes may therefore look at the relationship between cued song recall and language knowledge gains as a function of baseline language skills.

From a neuroscience perspective, it has been observed that the rhythmic organization of linguistic materials may be beneficial to the phonological encoding process in the human cognitive system (Cason and Schön, 2012). Moreover, based on the hypothesis of overlapping networks for syntactic processing of music and speech (Jentschke and Koelsch, 2009), some researchers have begun to explore the role of musical elements and music learning in speech acquisition with some emphasis on phonological awareness and reading fluency (see the meta-analysis by Gordon et al., 2015). However, there is clearly a need to address learning gains in writing through music and pay attention to the singular linguistic features in specific languages with their varying, and in some cases, idiosyncratic grammatical phenomena. Differing rhythmic patterns in languages also need to be considered, as recent research suggests that learners of German and other trochaic languages may particularly benefit from rhythmic accompaniment emphasizing the stressed syllable (see Schmidt-Kassow et al., 2018).

There are other limitations to this study, which need to be discussed. Firstly, in quasi-experimental studies, there are threats to internal validity through the nesting of students within groups and schools. However, as the recently migrated students explored in this study were recruited from different classes, nesting effects through long-lasting group membership are not likely. We also addressed the challenges of quasi-experimental studies by a rigorous research procedure which included baseline, post-, and follow-up tests, external teachers (instead of students' own teachers) and a standardized teaching script. Besides, relevant learning prerequisites (prior language knowledge, cognitive abilities) were gathered as potential control variables, and groups were compared with respect to these variables before the intervention.

Secondly, the length of the intervention was relatively short (three sessions/ 40 min. each). This was due to logistical reasons; schools had to allocate extra sessions and rooms to the intervention, which would make it difficult to conduct it for a more extended period of time. Students also still had to be accompanied to rooms, which required considerable time and staff resources from schools. However, the intervention was effective in light of its brevity and the heterogeneous group of learners. Given that significant learning progress occurred during the short intervention interval (2–3 weeks) and no significant learning progress occurred over the longer retention interval (3–4 weeks), it is unlikely that the observed effects were due to general speech development. Alternatively, future studies may control for developmental effects by the inclusion of a control group. In the present study, we refrained from including a control group for ethical reasons.

Thirdly, it was quite difficult to disentangle the effects of singing vs. speaking, as in all sessions, speaking and singing were combined. This design increased ecological validity, as singing is usually not employed without speaking in regular school lessons. One may assume that a positive motivational effect of singing carried over to the speaking modality (see above). In order to explore possible motivational effects, future studies should separate sessions where students either speak or sing. In addition, measurement of grammar and vocabulary gains through independent tasks may be useful to explore the two domains separately.

Despite these limitations, the results of our study indicate that the usefulness of songs for grammar teaching should be explored more in second language learning. Younger children, who tend to have short attention spans and more difficulties with explicit grammar instruction, may particularly benefit from songs geared at grammatical training. Given that singing does also not require any additional resources, results are of high practical value.

Using song in combination with or in addition to traditional teaching methods appears promising from both a linguistic and an educational perspective. Informal observational data and teachers accounts suggest that students were more concentrated and disciplined when they started singing. Singing together thus helped to share learning content without interruption of learning processes. Future studies should therefore also explore the effect of singing on classroom behavior and concentration. Overall, our study adds to the literature exploring the usefulness of singing in language education (e.g., Ludke et al., 2014; Good et al., 2015), suggesting, however, that it may not only be beneficial for pronunciation or vocabulary building, but also for promoting grammatical skills, which is still an under-researched area. Following the results by Stanat et al. (2012), who successfully used implicit and explicit teaching strategies with younger L2 learners, future studies could also explore to what extent additional grammar explanations could further enhance the effectivity of the songs. In that case, a sample of slightly more advanced migrant students should be chosen, as our current sample had very limited language proficiency. It should also be noted while students were not explicitly taught grammar rules, the songs used in the intervention were specifically tailored to convey grammatical patterns which were repeated throughout the songs.

As there was no clear difference between the two teaching modalities on cued recall of song lyrics and the singing and speaking modalities proved equally effective, results are not conclusive. Nevertheless, our study indicates that teaching vocabulary and grammar through song may be advantageous for recently migrated L2 learners in primary school. Given the general positive effect of music and singing on well-being (Clift et al., 2010) and cooperation (Good and Russo, 2016), the effect of learning through song with recently migrated students should be explored further.

## Author contributions

All authors listed have made a substantial, direct and intellectual contribution to the work, and approved it for publication.

### Conflict of interest statement

The authors declare that the research was conducted in the absence of any commercial or financial relationships that could be construed as a potential conflict of interest.

## Footnotes

^1^Unlike FL (foreign language) learners, who are usually not exposed to the target language outside the classroom, L2 (second language) learners are exposed to the target language outside the school context. Depending on the amount of exposure, learners' language proficiency can vary greatly.
