# Supplementary material for: Combining Song—And Speech-Based Language Teaching: An Intervention With Recently Migrated Children
Source: Front Psychol. 2018 Nov 28;9:2386. doi: 10.3389/fpsyg.2018.02386 (PMC6279872; doi:10.3389/fpsyg.2018.02386)
Supplement: Supplementary file 1 [file Data_Sheet_1.DOCX]

**Appendix A**

Song A: Wen magst du?

Der **Bauer** mag die **Bäuerin**.

Der **Bäcker** mag die **Bäckerin**.

Der **Lehrer** mag die **Lehrerin**.

Und wen **magst** du?

Der **Jäger** mag die **Jägerin**.

Der **Maler** mag die **Malerin**.

Der **Schneider** mag die **Schneiderin**.

Und wen **magst** du?

Der **Schüler** mag die **Schülerin**.

Der **Sportler** mag die **Sportlerin**.

Der **Tänzer** mag die **Tänzerin**.

Und ich **mag** dich!

**Appendix B**

Song B: Mitmach-Lied

Ich **wackle** mit dem **Kopf**.

Wir **wackeln** mit den **Köpfen**.

Ich **trommle** auf den **Topf**.

Wir **trommeln** auf den **Töpfen**.

**Kopf** – **Köpfe**, **Topf** – **Töpfe**.

Ich **klatsche** in die **Hand**.

Wir **klatschen** in die **Hände**.

Ich **klopfe** an die **Wand**.

Wir **klopfen** an die **Wände**.

**Hand** – **Hände**, **Wand** – **Wände**.

Ich **laufe** durch den **Raum**.

Wir **laufen** durch die **Räume**.

Ich **stehe** wie ein **Baum**.

Wir **stehen** wie die **Bäume**.

**Raum** – **Räume**, **Baum** – **Bäume**.

Appendix C

**Pearson correlations for language test within t1**

| **Variable and time (t1)** | **1** | **2** | **3** | **4** | **5** | **6** |
| --- | --- | --- | --- | --- | --- | --- |
| 1. vocabulary knowledge (matching verbs and nouns to pictures; 12 items) | - | .49** | .69** | .63** | .44** | .65** |
| 2. verb conjugation I (choosing the correct personal pronoun for a given conjugated verb; 6 items) | .49** | - | .38* | .57** | .62** | .76** |
| 3. verb conjugation II (conjugating verbs based on a given personal pronoun; 6 items) | .69** | .38* | - | .55** | .50** | .71** |
| 4. plural forms (providing the correct plural form of a given noun; 4 items) | .63** | .57** | .55** | - | .54** | .74** |
| 5. feminine ending of agent nouns (modifying a given job title to the female form) (4 items) | .44** | .62** | .50** | .54** | - | .86** |
| 6. transfer items (application of grammatical rules to unknown nouns and verbs) | .65** | .76** | .71** | .74** | .86** | - |

*Note*. * Correlations significant at the 0.05 level (two-tailed). ** Correlations significant at the 0.01 level (two-tailed). *N* = 35.

**Pearson correlations for language test within t2**

| **Variable and time (t2)** | **1** | **2** | **3** | **4** | **5** | **6** |
| --- | --- | --- | --- | --- | --- | --- |
| 1. vocabulary knowledge (matching verbs and nouns to pictures; 12 items) | - | .33 | .34* | .53** | .69** | .61** |
| 2. verb conjugation I (choosing the correct personal pronoun for a given conjugated verb; 6 items) | .33 | - | .27 | .30 | .56** | .69** |
| 3. verb conjugation II (conjugating verbs based on a given personal pronoun; 6 items) | .34* | .27 | - | .53** | .55** | .67** |
| 4. plural forms (providing the correct plural form of a given noun; 4 items) | .53** | .30 | .53** | - | .61** | .71** |
| 5. feminine ending of agent nouns (modifying a given job title to the female form) (4 items) | .69** | .56** | .55** | .61** | - | .87** |
| 6. transfer items (application of grammatical rules to unknown nouns and verbs) | .61** | .69** | .67** | .71** | .87** | - |

*Note*. * Correlations significant at the 0.05 level (two-tailed). ** Correlations significant at the 0.01 level (two-tailed). *N* = 35.

**Pearson correlations for language test within t3**

| **Variable and time (t3)** | **1** | **2** | **3** | **4** | **5** | **6** |
| --- | --- | --- | --- | --- | --- | --- |
| 1. vocabulary knowledge (matching verbs and nouns to pictures; 12 items) | - | .28 | .40* | .35* | .41* | .42* |
| 2. verb conjugation I (choosing the correct personal pronoun for a given conjugated verb; 6 items) | .28 | - | .43* | .59** | .45** | .76** |
| 3. verb conjugation II (conjugating verbs based on a given personal pronoun; 6 items) | .40* | .43* | - | .54** | .56** | .78** |
| 4. plural forms (providing the correct plural form of a given noun; 4 items) | .35* | .59** | .54** | - | .36* | .68** |
| 5. feminine ending of agent nouns (modifying a given job title to the female form) (4 items) | .41** | .45** | .56** | .36* | - | .73** |
| 6. transfer items (application of grammatical rules to unknown nouns and verbs) | .42* | .76** | .78** | .68** | .73** | - |

*Note*. * Correlations significant at the 0.05 level (two-tailed). ** Correlations significant at the 0.01 level (two-tailed). *N* = 35.

**Pearson correlations for language test between t1 and t2; t2 and t3**

| **Variable and time (t1/t2 and t2/t3)** |  |  | |
| --- | --- | --- | --- |
|  | t1/t2 | | t2/t3 |
| 1. vocabulary knowledge (matching verbs and nouns to pictures; 12 items) | .57** | | .74** |
| 2. verb conjugation I (choosing the correct personal pronoun for a given conjugated verb; 6 items) | .59** | | .52** |
| 3. verb conjugation II (conjugating verbs based on a given personal pronoun; 6 items) | .49** | | .64** |
| 4. plural forms (providing the correct plural form of a given noun; 4 items) | .58** | | .78** |
| 5. feminine ending of agent nouns (modifying a given job title to the female form) (4 items) | .58** | | .66** |
| 6. transfer items (application of grammatical rules to unknown nouns and verbs; 9 items) | .81** | | .74** |

*Note*. ** Correlations significant at the 0.01 level (two-tailed). *N* = 35.
